# Acute administration of interleukin‐6 does not increase secretion of glucagon‐like peptide‐1 in mice

**DOI:** 10.14814/phy2.13788

**Published:** 2018-07-06

**Authors:** Charlotte B. Christiansen, Sara J. Lind, Berit Svendsen, Emilie Balk‐Møller, Tina Dahlby, Rune E. Kuhre, Bolette Hartmann, Thomas Mandrup‐Poulsen, Carolyn F. Deacon, Nicolai J. Wewer Albrechtsen, Jens J. Holst

**Affiliations:** ^1^ Department of Biomedical Sciences Faculty of Health and Medical Sciences University of Copenhagen Copenhagen Denmark; ^2^ NNF Center for Basic Metabolic Research Faculty of Health and Medical Sciences University of Copenhagen Copenhagen Denmark; ^3^ Department of Clinical Biochemistry, Rigshospitalet University of Copenhagen Copenhagen Denmark

**Keywords:** GLP‐1, glucagon, glucose, IL‐6, insulin

## Abstract

Interleukin 6 (IL‐6) is a cytokine secreted from skeletal muscle in response to exercise which, based on animal and cell studies, has been suggested to contribute to glucose metabolism by increasing secretion of the incretin hormone glucagon‐like peptide‐1 (GLP‐1) and affecting secretion of insulin and glucagon from the pancreatic islets. We investigated the effect of IL‐6 on GLP‐1 secretion in GLP‐1 producing cells (GLUTag) and using the perfused mouse small intestine (harboring GLP‐1 producing cells). Furthermore, the direct effect of IL‐6 on insulin and glucagon secretion was studied using isolated perfused mouse pancreas. Incubating GLUTag cells with 1000 ng/mL of IL‐6 for 2 h did not significantly increase secretion of GLP‐1 whereas 10 mmol/L glucose (positive control) did. Similarly, IL‐6 (100 ng/mL) had no effect on GLP‐1 secretion from perfused mouse small intestine whereas bombesin (positive control) increased secretion. Finally, administering IL‐6 (100 ng/mL) to perfused mouse pancreases did not significantly increase insulin or glucagon secretion regardless of perfusate glucose levels (3.5 vs. 12 mmol/L glucose). Acute effects of IL‐6 therefore do not seem to include a stimulatory effect on GLP‐1 secretion in mice.

## Introduction

Interleukin‐6 (IL‐6) is a cytokine, secreted from immune cells and other cells, which has both pro‐inflammatory and anti‐inflammatory effects (Fischer 2006; Scheller et al. 2011). Previous studies have linked IL‐6 to metabolic diseases, including type 2 diabetes (Pedersen et al. 2001). However, the role of IL‐6 in regulation of glucose metabolism is not well‐characterized (Xu et al. 2003; Fischer 2006; Ellingsgaard et al. 2008; Timper et al. 2016). Conflicting evidence regarding both harmful and beneficial effects of IL‐6 have been reported: thus in humans, adipose tissue derived IL‐6 was found to contribute to insulin resistance in type 2 diabetes, whereas skeletal muscle derived IL‐6, secreted in response to an acute bout of exercise, may be associated with improved beta‐cell function and insulin sensitivity (Ostrowski et al. 1998; Steensberg et al. 2000; Carey et al. 2006). Recent animal and cell studies have suggested that acute IL‐6‐induced insulin secretion (IL‐6 exposure below 24 h) involves a direct effect on enteroendocrine l‐cells, resulting in an increased secretion of the incretin hormone, glucagon‐like peptide‐1 (GLP‐1) (Ellingsgaard et al. 2011; Kahles et al. 2014; Wueest et al. 2018). GLP‐1 is normally secreted from l‐cells in response to nutrient ingestion and binds to its cognate receptor expressed on pancreatic beta‐cells, leading to potentiation of glucose‐induced insulin secretion (insulinotropic effects); it also inhibits the secretion of glucagon (glucagonostatic effects) (Hare et al. 2010) and through this combined action, may lower hepatic glucose production and decrease plasma glucose concentrations (Hvidberg et al. 1994). Today, these effects are utilized in the treatment of type 2 diabetes with GLP‐1 receptor agonists (Drucker et al. 2017).

We were therefore interested in knowing more about IL‐6‐mediated regulation of GLP‐1 secretion, and investigated the acute effect of exogenous IL‐6 in perfused mouse small intestine and in cultured GLP‐1 producing cells. The potential direct effects of IL‐6 on insulin and glucagon secretion were then studied using the perfused mouse pancreas.

## Methods

### Ethical approvals

Handling of the animals was performed in accordance with internationally accepted guidelines and with permission from the Danish Animal Experiments Inspectorate (license no. 2013‐15‐2934‐00833).

### Perfusion studies

Female C57BL/6JRj mice (Janvier, Saint Berthevin Cedex, France) fed ad libitum and weighing between 25 and 30 g were used as donors. Animals were housed under a 12:12 h light‐dark cycle and after approximately 1 week of acclimatization they were used for experiments. The mice were anesthetized with an intraperitoneal injection of ketamine/xylazine (Ketamine 90 mg/kg (Ketaminol Vet.; MSD Animal Health, Madison, NJ, USA) + xylazine 10 mg/kg (Rompun Vet.; Bayer Animal Health, Leverkusen, Germany) before surgery.

After lack of reflexes was established, the pancreas or the proximal small intestine was isolated and perfused in situ as described previously (Svendsen et al. 2016; Orgaard and Holst 2017). Briefly, the pancreas or the proximal half of the small intestine was perfused in a single‐pass system through a catheter inserted into the supplying abdominal aorta. All other aortic branches were ligated. The venous effluent was collected for 1 min intervals via a draining catheter inserted into the portal vein, and stored at −20°C until analysis. We used equipment dedicated for rodent organ perfusion (Hugo Sachs Elektronik, March‐Hugstetten, Germany), and the flow rate was kept constant at 1.5 mL/min (pancreas perfusion) or 2.5 mL/min (small intestine perfusion). The perfusion medium consisted of a modified Krebs‐Ringer bicarbonate buffer containing in addition 5% dextran T‐70 (Pharmacosmos, Holbæk, Denmark), 0.1% bovine serum albumin (Faction V, Merck, Ballerup, Denmark), 3.5 mmol/L glucose, and 5 mmol/L pyruvate, fumarate and glutamate. The perfusion medium was heated to 37°C and continuously gassed throughout the experiment with 95% O_2_ and 5% CO_2_. Prior to protocol start, the organ was perfused with the basal perfusion medium for a 30 min equilibrium period. Mouse IL‐6 protein (R&D Systems, Minneapolis, USA, cat.no. 406‐ML‐200) was dissolved in PBS containing 0.1% human serum albumin and further diluted in perfusion buffer. IL‐6 was infused through a sidearm syringe infusion pump into the arterial supply of the pancreas or the proximal small intestine to reach a final concentration of 100 ng/mL. l‐Arginine monohydrochloride (10 mmol/L) (Sigma‐Aldrich, Steinheim, Germany, cat.no. A6969) dissolved in perfusion buffer was used as a positive control to the perfused pancreas. Bombesin (Bachem, Bubendorf, Switzerland, cat.no. H‐2155) was dissolved in dimethyl sulfoxide and perfusion buffer and added in a final concentration of 10 nmol/L as a positive control in the perfused small intestine.

### Cell studies

GLUTag cells were maintained at 37°C, 5% CO_2_ and grown in low glucose (1.0 g/L) DMEM supplemented with 10% (v/v) fetal bovine serum (FBS), 1% (v/v) penicillin/streptomycin and 1% (v/v) l‐glutamine. Cells were kept until 70–80% confluent and then split and plated on 24 well plates precoated with matrigel (1:100; cat.no. 354234; BD Biosciences, Bedford, MA). The following day, cells were examined for visual appearance and proper monolayer formation. Cells (~75% confluent) were thoroughly washed with PBS and incubated with 250 *μ*L test substance for 2 h (at 37°C, 5% CO_2_). Test substances consisted of mouse IL‐6 (100 and 1000 ng/mL, R&D Systems, Minneapolis, USA, cat.no. 406‐ML‐200), or 10 mmol/L glucose (positive control) diluted in same bath solution as baseline. Supernatants were collected and centrifuged (1500*g*, 5 min, room temperature) to remove potential floating cells or cell debris. Samples were stored at −20°C until analysis.

The A20 mouse B‐lymphocyte cell line (A gift from S. Buus, Department of Immunology and Microbiology, University of Copenhagen, Denmark) was maintained in complete medium (RPMI 1640 GlutaMAX (cat.no. 61870‐010) supplemented with 10% FBS (cat.no. 26140‐079), 1% penicillin/streptomycin (cat.no 15140‐122), 10 mmol/L HEPES (cat.no. 15630‐056), 1 mmol/L sodium pyruvate (cat.no. 11360‐070), 50 *μ*mol/L *β*‐mercaptoethanol (cat.no. 31350‐010), 4500 mg/L d‐glucose (cat.no. A24940‐01) [all from Life Technologies, Paisley, United Kingdom]) at 37°C in a humidified atmosphere containing 5% CO_2_. Cells were passaged every 3 days and precultured for 24 h prior to experimental procedure. Experiments were performed within passage numbers 25–28.

### STAT3 phosphorylation assay

Two million A20 mouse B‐cells were seeded in 35 mm culture dishes (VWR, Søborg, Denmark) in 2 mL complete medium. After 24 h of preculture, cells were collected in Eppendorf tubes, briefly centrifuged at 100 rpm, gently resuspended in 2 mL Krebs‐Ringer bicarbonate buffer containing 100 ng/mL IL‐6 (positive control), no IL‐6 (negative control) or perfusion samples (#1‐#4). The four perfusion samples were pooled effluents collected from four different perfusion experiments (4 mice) during infusion of IL‐6. Cells were plated on 35 mm culture and incubated at 37°C, 5% CO_2_. After one hour, cells were collected in Eppendorf tubes, centrifuged (153 *g*, 10 min, 4°C), and the pellet was lysed in lysis buffer (50 mmol/L Tris pH 8, 150 mmol/L NaCl, 5 mmol/L KCl, 5 mmol/L MgCl_2_, 0.5% NP‐40) supplemented with protease (Roche, Mannheim, Germany, cat.no. 11 836 153 001) and phosphatase inhibitors (Roche, Mannheim, Germany, cat.no. 04 906 845 001). After 30 min on ice, lysates were centrifuged (20,817 *g*, 20 min, 4°C) and supernatants were stored at −80°C. Protein concentrations in lysates were determined using Bradford Reagent (Bio‐Rad, cat.no. 5000006) to adjust for protein concentration. Samples were prepared in Laemmli Sample Buffer (Bio‐Rad, cat.no. 161‐0747) with *β*‐mercaptoethanol (Sigma‐Aldrich, Steinheim, Germany, cat.no. M3148), protein separated on NuPAGE™ 4–12% Bis‐Tris Protein Gels (Life Technologies, Paisley, United Kingdom, cat.no. NP0336BOX) and transferred to PVDF membranes (Life Technologies, Paisley, United Kingdom, cat.no. IB24002). Membranes were blocked in 5% bovine serum albumin (BSA, Sigma‐Aldrich, Steinheim, Germany, cat.no. A7906) in Tris‐buffered saline (50 mmol/L Tris, 150 mmol/L NaCl; TBS). Primary antibodies were diluted in 2.5% BSA in TBS + 0.1% Tween (TBST) and incubated with membranes overnight at 4°C. Secondary HRP‐conjugated antibodies were diluted 1:10,000 in 5% nonfat milk in TBST. Blots were developed using a chemiluminescence detection system (Clarity™ Western ECL Substrate, Bio‐Rad, cat.no. 170‐5061) and the light emission was captured using a Syngene G:BOX system (Syngene, Cambridge, United Kingdom).

Antibodies used: anti‐Phospho‐STAT3 (Cell Signaling, USA, cat.no. 9131) at 1:1000, anti‐STAT3 (Cell Signaling, USA, cat.no. 4904) at 1:2000, anti‐*α*‐tubulin (Sigma‐Aldrich, Steinheim, Germany, cat.no. T 6047) at 1:10,000, anti‐rabbit IgG HRP‐linked antibody (Cell Signaling, USA, cat.no. 7074), anti‐mouse IgG HRP‐linked antibody (Cell Signaling, USA, cat.no. 7076).

### Biochemical measurements

Total GLP‐1 concentrations (the sum of GLP‐1 1–36NH_2_, 7–36NH_2_ and 9–36NH_2_) were determined using an in‐house radioimmunoassay (RIA) (codename 89390) (Ørskov et al. 1994; Wewer Albrechtsen et al. 2015). Insulin concentrations were determined using an in‐house RIA (codename 2006‐3) that cross‐reacts with both human, rat and mouse insulin (Brand et al. 1995). Glucagon was measured using a C‐terminally directed antiserum (codename 4305), which measures fully processed glucagon (Orskov et al. 1991).

Presence of IL‐6 protein in infusion solutions and effluent perfusion samples was measured by ELISA (R&D Systems, Minneapolis, USA, cat.no M6000B), carried out according to the protocol supplied by the manufacturer. Each sample was measured using different dilutions and in duplicates.

### Statistical analysis

To assess distribution and homoscedasticity in datasets, the Shapiro–Wilk test (swilk command) was applied and residual plots were drafted. Areas under the curves (AUC) were calculated using the trapezoidal rule. For perfusion studies, baseline was defined as 5 min before stimulation and 5 min before the next stimulation. Student *t*‐test was used to assess differences between two groups whereas one‐way ANOVA, corrected by a post hoc analysis (Sidak) for multiple testing, was used for testing differences between more than two groups of data. Power calculation was performed in order to allow an alpha value of 0.05 (two‐sided), with an effect size of 20% and a beta value of ~0.90.

Calculations were made using GraphPad Prism version 6.04 for Windows, GraphPad Software, La Jolla California USA, http://www.graphpad.com and STAT14 (SE), College Station, Texas 77845, USA. For illustrations the Adobe CC software suite was used (San Francisco, CA 94103, USA).

## Results

### Acute administration of IL‐6 to isolated perfused mouse small intestine and GLP‐1 producing GLUTag cells did not significantly increase secretion of GLP‐1

**Figure 1:**
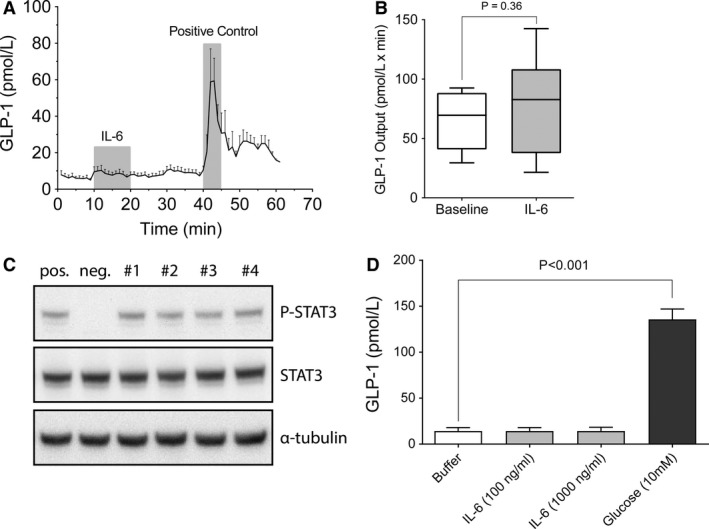
Lack of effect of IL‐6 on GLP‐1 Secretion in the Perfused Mouse Small Intestine and in GLP‐1 producing GLUTag cells. (A) GLP‐1 secretion (pmol/L) in perfused mouse small intestines (*n* = 6). Infusion of IL‐6 (100 ng/mL) (10–20 min), followed by a period in the absence of IL‐6 (21–39 min), and finally, a period in the presence of 10 nmol/L of bombesin (positive control; 40–45 min). (B) Hormone output (secretion) calculated as area under the curve, with (gray) or without (white) IL‐6 (100 ng/mL), was calculated for each mouse and illustrated collectively as bar and whiskers (Tukey distribution). (C) IL‐6 bioactivity in perfusion samples (#1‐#4) was determined by the ability of these perfusates to induce STAT3 phosphorylation (P‐STAT3) as analyzed by SDS‐PAGE and Western blotting. One‐hundred ng/mL of mIL‐6 was used as positive control (pos.) and Krebs‐Ringer bicarbonate buffer as negative control (neg.). Total STAT3 and *α*‐tubulin were used as loading controls. (D) GLP‐1 levels in cell media after 2 h incubation with buffer, 100 or 1000 ng/mL IL‐6, or 10 mmol/L glucose (*n* = 4). IL‐6 had no effect on GLP‐1 secretion compared to basal levels in either of these experimental models. Data in panel A and D are shown as mean ± SEM and in panel B as box and whisker (Tukey distribution).

To address whether elevated circulating IL‐6 has an impact on enteroendocrine GLP‐1 secretion, we infused recombinant IL‐6 protein (100 ng/mL) into the arterial supply of the in situ perfused proximal mouse small intestine (*n* = 6). Addition of IL‐6 had no significant impact on GLP‐1 secretion (Fig. 1A and B, *P* = 0.36), whereas secretion was significantly (*P* < 0.01) increased after administration of 10 nmol/L bombesin (phospholipase C activator, positive control). To verify that the IL‐6 which was infused in these experiments was still bioactive during the perfusion, we used the phosphorylation of Signal Transducer and Activator of Transcription 3 (STAT3) as a bioassay, by incubating mouse B‐lymphocytic A20 cells with perfusion effluents from same experiments as shown in Figure 1A and B. Protein levels of phosphorylated STAT3, but not STAT3, were increased in all of the samples tested from four mice compared to buffer alone (Fig. 1C).

GLP‐1 producing (GLUTag cell line) cells were incubated for 2 h with mouse IL‐6 protein (100 or 1000 ng/mL) to further evaluate any effect of IL‐6 on GLP‐1 secretion (*n* = 4). GLP‐1 secretion was not significantly affected by IL‐6 administration (*P* > 0.90) in either group compared to buffer alone (Fig. 1D). In contrast, 10 mmol/L glucose (positive control) led to a ~ninefold increase in GLP‐1 secretion (*P *< 0.001).

### Acute administration of IL‐6 to isolated perfused mouse pancreases did not significantly increase secretion of insulin or glucagon

**Figure 2:**
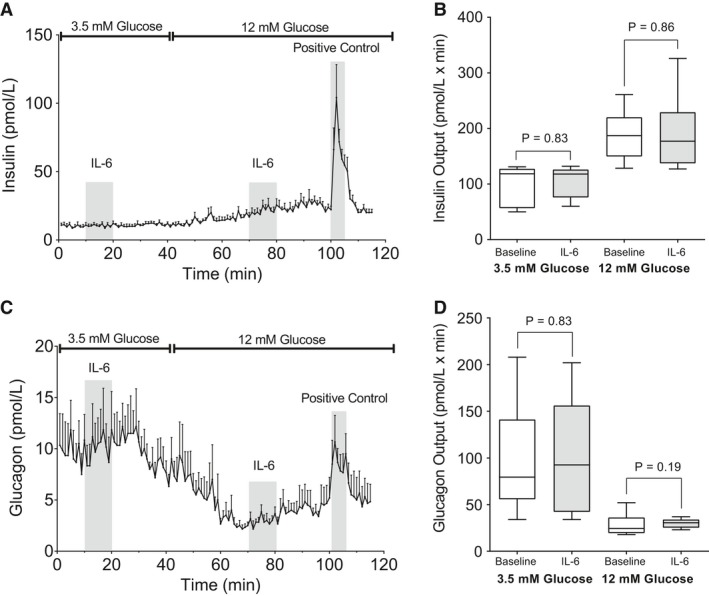
The effect of IL‐6 on Insulin and Glucagon Secretion in the Perfused Mouse Pancreas. (A) Insulin secretion (pmol/L) from perfused mouse pancreases (*n* = 6). IL‐6 (100 ng/mL) was infused for 10 min (10–20 min) during perfusion with medium containing 3.5 mmol/L glucose (0–39 min). The infusion was repeated (at 70–80 min) during perfusion with 12 mmol/L glucose in the medium. Finally, 10 mmol/L of l‐arginine (positive control) was administered during perfusion with 12 mmol/L glucose (100–105 min). (B) Insulin output (secretion), calculated as area under the curve, with (gray) or without (white) IL‐6 (100 ng/mL) as shown in panel A, was calculated for each mouse during low (3.5 mmol/L) and high (12 mmol/L) glucose, and shown as bar and whiskers (tukey). (C) Glucagon secretion (pmol/L) from perfused mouse pancreases (*n* = 6) in the same experiments as those shown in panel A. (D) Glucagon output (secretion) calculated as area under the curve from the data shown in panel C. IL‐6 did not change insulin or glucagon secretion from basal levels in perfused mouse pancreases. Data in panel A and C are shown as mean ± SEM and in panel B and D as box and whisker (Tukey distribution).

The acute effect of IL‐6 (100 ng/mL) on insulin and glucagon secretion was examined at both low (3.5 mmol/L) and high (12 mmol/L) glucose levels using the in situ perfused mouse pancreas (*n* = 6). Pancreatic insulin secretion was unaffected by IL‐6 infusion in the presence of low (*P* = 0.83) or high (*P* = 0.86) glucose, whereas insulin secretion increased 3‐fold (*P* = 0.02) in the presence of high glucose compared to low glucose, as expected (Fig. 2A and B).

Glucagon secretion was not influenced by IL‐6 infusion at either 3.5 (*P* = 0.96) or 12 mmol/L glucose concentration (*P* = 0.19) (Fig. 2C and D). Raising the glucose concentration to 12 mmol/L inhibited glucagon secretion 5‐fold (*P* = 0.03).

Administering 10 mmol/L arginine (positive control) in combination with 12 mmol/L glucose significantly (*P* < 0.05) stimulated both insulin and glucagon secretion at the end of the experiments.

## Discussion

The present studies were carried out to elucidate the possible effects of IL‐6 on glucose metabolism in more detail, with a focus on its effects on endocrine secretion from the gut and the pancreas, inspired by the findings reported by Ellingsgaard et al. (2011). However, in the present study, we were unable to demonstrate a significant *acute* effect of IL‐6 on GLP‐1 secretion from GLP‐1 producing cells (GLUTag) or from perfused mouse small intestine.

We measured IL‐6 concentrations in all in vitro experiments in order to assess whether IL‐6 was degraded or whether lower than calculated amounts of IL‐6 had been added. However, concentrations of IL‐6 were always within ±10% of the expected concentrations. Furthermore, using phosphorylation of STAT3 induced by IL‐6 containing effluents as bioassay, we prove that the IL‐6 administered was biologically active and not degraded during the perfusion (Harder‐Lauridsen et al. 2014). The concentrations of IL‐6 used in this study (100 or 1000 ng/mL) were chosen based on levels previously reported to result in increased GLP‐1 secretion (Ellingsgaard et al. 2011; Kahles et al. 2014; Wueest et al. 2018). On the other hand, it was impossible, within the framework of the current experiments, to study effects of prolonged, chronic administration of IL‐6 (more than 24 h), which would have been relevant, given the reported up‐regulation by IL‐6 of proglucagon expression in the gut (Ellingsgaard et al. 2011). Furthermore, another theoretical limitation might be that we did not include an additional stimulus for GLP‐1 secretion (e.g. costimulation with glucose). However, the perfusion buffer does contain some glucose (~3.5 mmol/L), and the preparation has a significant basal secretion of GLP‐1. Moreover, bombesin, added as a positive control, did markedly stimulate secretion, suggesting that the intracellular machinery for GLP‐1 secretion was activated and able to respond to additional stimulation. It seems unlikely that any increase in GLP‐1 secretion escaped detection since the GLP‐1 assay we used is very sensitive and has been validated in several studies (Bak et al. 2014; Wewer Albrechtsen et al. 2015). It is directed toward the amidated C‐terminus of the GLP‐1 molecule which is appropriate since, in the mouse, nearly all extractable GLP‐1 is amidated (Kuhre et al. 2014). Moreover, by using a C‐terminal assay, all molecular forms carrying the amidation would be detected, including any extended or degraded molecular forms (Windeløv et al. 2017). In addition, using the isolated perfused mouse intestine allowed us to determine any effect on secretion with much greater sensitivity compared to what would have been possible in in vivo studies in mice (Windeløv et al. 2017).

Previous human studies have shown ambiguous insulin responses to IL‐6, since IL‐6 administration increased insulin‐stimulated glucose disposal in a study by Carey et al. (2006), but decreased insulin secretion relative to placebo in a study by Harder‐Lauridsen et al. (2014). However, the first study was performed in healthy subjects, while the latter was performed in subjects with type 2 diabetes, which might explain the opposing outcomes, as the latter are less sensitive to insulin (Vilsboll and Holst 2004; Holst et al. 2011). In the present experiments, IL‐6 neither increased nor decreased insulin secretion, at least not acutely via a direct mechanism. Similarly, pancreatic glucagon secretion appears not to be under the direct control of circulating IL‐6, in contrast with earlier reports describing increased alpha‐cell derived glucagon secretion from human islets upon IL‐6 incubation (Ellingsgaard et al. 2008). In a recently published human study, there was no acute stimulatory effect of IL‐6 on GLP‐1 secretion, but IL‐6 did seem to improve glycemic tolerance. This, however, was considered to be related to a delayed gastric emptying, independent of GLP‐1 receptor signaling (Lehrskov et al. 2018). High expression of the IL‐6 receptor in the endocrine pancreas, especially on alpha‐cells, has been reported in both mice and rats (Ellingsgaard et al. 2008), but our data do not support that activation of this receptor impacts glucagon secretion, at least not in an acute manner in mice.

Our data do not support that IL‐6 stimulates GLP‐1 secretion from the mouse intestine, and therefore also question the existence of an IL‐6 ‐induced cross‐talk between the gut, the endocrine pancreas and insulin sensitive tissues. Rather, the present findings are in line with a recent study demonstrating no GLP‐1 response to IL‐6 in individuals with type 2 diabetes (Harder‐Lauridsen et al. 2014). In the intestine, GLP‐1 is generated by PC1/3 processing of proglucagon while, in the pancreatic alpha cells, proglucagon is cleaved by PC2, resulting in the formation of glucagon (Deacon and Holst 2009). Small amounts of two other N‐terminally extended GLP‐1 isoforms (inactive) may also be formed in the pancreas (GLP‐1(1–37) and GLP‐1(1–36 _NH2_)) (Holst 1997). Theoretically, in vivo, an effect of IL‐6 on GLP‐1 plasma levels could be due to stimulation of the alpha cells. However, our results also indicate that IL‐6 does not directly affect the endocrine pancreas. Reprogramming of pancreatic alpha‐cells to express PC1/3, and hence to be capable of secreting active GLP‐1, has attracted great interest (Ellingsgaard et al. 2011). It cannot, therefore, be excluded that IL‐6 might provoke such reprogramming, and given the time required for this to happen, it would not be possible for us to observe this effect in our experiments. However, to date, we have not been able to detect active GLP‐1 in the pancreas of healthy mice (Galsgaard et al. 2018).

Immune cells and adipocytes are among the various tissues that produce and secrete IL‐6 protein (Fischer 2006). In addition, Pedersen et al. have shown that IL‐6 is acutely secreted from human skeletal muscle during intensive exercise and is associated with improved insulin sensitivity (Ostrowski et al. 1998; Steensberg et al. 2000; Carey et al. 2006). Similarly, muscle damage induced by exhaustive exercise is associated with production of inflammatory cytokines, leading to recruitment of immune cells responsible for postexercise repair mechanisms (Ostrowski et al. 1998; Steensberg et al. 2003a). During rest, increased insulin‐mediated glucose uptake was found in healthy men after IL‐6 administration (Carey et al. 2006). However, other studies in both individuals with type 2 diabetes and healthy subjects found no change in insulin‐mediated glucose uptake or plasma glucose concentrations following IL‐6 administration (Steensberg et al. 2003b; Harder‐Lauridsen et al. 2014). Taken together, these studies raise the possibility that IL‐6 could have differential roles in glucose homeostasis, one depending on long‐term effects of IL‐6 receptor activation (potentially mediated by adaptation of proglucagon expression in l‐and alpha‐cells) and another acting independently of the incretin‐axis.

In conclusion, an acute direct effect of IL‐6 on GLP‐1, insulin and glucagon secretion was not observed in this study. Future studies investigating chronic effects of IL‐6 on GLP‐1 secretion in humans are therefore warranted.

## Conflict of Interest

We have no conflicts of interest to declare.

## References

[phy213788-bib-0002] Bak, M. J. , N. J. Wewer Albrechtsen , J. Pedersen , F. K. Knop , T. Vilsbøll , N. B. Jørgensen , et al. 2014 Specificity and sensitivity of commercially available assays for glucagon‐like peptide‐1 (GLP‐1): implications for GLP‐1 measurements in clinical studies. Diabetes Obes. Metab. 16:1155–1164. 25041349 10.1111/dom.12352

[phy213788-bib-0003] Brand, C. L. , P. N. Jorgensen , U. Knigge , J. Warberg , I. Svendsen , J. S. Kristensen , et al. 1995 Role of glucagon in maintenance of euglycemia in fed and fasted rats. Am. J. Physiol. Endocrinol. Metab. 269:E469–E477. 10.1152/ajpendo.1995.269.3.E469 7573424

[phy213788-bib-0004] Carey, A. L. , G. R. Steinberg , S. L. Macaulay , W. G. Thomas , A. G. Holmes , G. Ramm , et al. 2006 Interleukin‐6 increases insulin‐stimulated glucose disposal in humans and glucose uptake and fatty acid oxidation in vitro via AMP‐activated protein kinase. Diabetes 55:2688–2697. 17003332 10.2337/db05-1404

[phy213788-bib-0005] Deacon, C. F. , and J. J. Holst . 2009 Immunoassays for the incretin hormones GIP and GLP‐1. Best Pract. Res. Clin. Endocrinol. Metab. 23:425–432. 19748060 10.1016/j.beem.2009.03.006

[phy213788-bib-0006] Drucker, D. J. , J. F. Habener , and J. J. Holst . 2017 Discovery, characterization, and clinical development of the glucagon‐like peptides. J. Clin. Invest. 127:4217–4227. 29202475 10.1172/JCI97233 PMC5707151

[phy213788-bib-0007] Ellingsgaard, H. , J. A. Ehses , E. B. Hammar , L. Van Lommel , R. Quintens , G. Martens , et al. 2008 Interleukin‐6 regulates pancreatic alpha‐cell mass expansion. Proc. Natl Acad. Sci. USA 105:13163–13168. 18719127 10.1073/pnas.0801059105 PMC2529061

[phy213788-bib-0008] Ellingsgaard, H. , I. Hauselmann , B. Schuler , A. M. Habib , L. L. Baggio , D. T. Meier , et al. 2011 Interleukin‐6 enhances insulin secretion by increasing glucagon‐like peptide‐1 secretion from L cells and alpha cells. Nat. Med. 17:1481. 22037645 10.1038/nm.2513 PMC4286294

[phy213788-bib-0009] Fischer, C. P. 2006 Interleukin‐6 in acute exercise and training: what is the biological relevance? Exerc. Immunol. Rev 12:6–33. 17201070

[phy213788-bib-0010] Galsgaard, K. D. , M. Winther‐Sørensen , C. Ørskov , H. Kissow , S. S. Poulsen , H. Vilstrup , et al. 2018 Disruption of glucagon receptor signaling causes hyperaminoacidemia exposing a possible liver‐alpha‐cell axis. Am. J. Physiol. Endocrinol. Metab. 314:E93–E103. 28978545 10.1152/ajpendo.00198.2017 PMC6048389

[phy213788-bib-0011] Harder‐Lauridsen, N. M. , R. Krogh‐Madsen , J. J. Holst , P. Plomgaard , L. Leick , B. K. Pedersen , et al. 2014 Effect of IL‐6 on the insulin sensitivity in patients with type 2 diabetes. Am. J. Physiol. Endocrinol. Metab. 306:E769–E778. 24473436 10.1152/ajpendo.00571.2013

[phy213788-bib-0012] Hare, K. J. , T. Vilsbøll , M. Asmar , C. F. Deacon , F. K. Knop , and J. J. Holst . 2010 The glucagonostatic and insulinotropic effects of glucagon‐like peptide 1 contribute equally to its glucose‐lowering action. Diabetes 59:1765–1770. 20150286 10.2337/db09-1414 PMC2889777

[phy213788-bib-0013] Holst, J. J. 1997 Enteroglucagon. Annu. Rev. Physiol. 59:257–271. 9074764 10.1146/annurev.physiol.59.1.257

[phy213788-bib-0014] Holst, J. J. , F. K. Knop , T. Vilsbøll , T. Krarup , and S. Madsbad . 2011 Loss of incretin effect is a specific, important, and early characteristic of type 2 diabetes. Diabetes Care 34(Suppl 2):S251–S257. 21525464 10.2337/dc11-s227 PMC3632188

[phy213788-bib-0015] Hvidberg, A. , M. T. Nielsen , J. Hilsted , C. Ørskov , and J. J. Holst . 1994 Effect of glucagon‐like peptide‐1 (proglucagon 78‐107amide) on hepatic glucose production in healthy man. Metab.‐Clin. Exp. 43:104–108. 8289665 10.1016/0026-0495(94)90164-3

[phy213788-bib-0016] Kahles, F. , C. Meyer , J. Möllmann , S. Diebold , H. M. Findeisen , C. Lebherz , et al. 2014 GLP‐1 secretion is increased by inflammatory stimuli in an IL‐6–dependent manner, leading to hyperinsulinemia and blood glucose lowering. Diabetes 63:3221–3229. 24947356 10.2337/db14-0100

[phy213788-bib-0017] Kuhre, R. E. , N. W. Albrechtsen , J. A. Windeløv , B. Svendsen , B. Hartmann , and J. J. Holst . 2014 GLP‐1 amidation efficiency along the length of the intestine in mice, rats and pigs and in GLP‐1 secreting cell lines. Peptides 55:52–57. 24486427 10.1016/j.peptides.2014.01.020

[phy213788-bib-0018] Lehrskov, L. L. , M. P. Lyngbaek , L. Soederlund , G. E. Legaard , J. A. Ehses , S. E. Heywood , et al. 2018 Interleukin‐6 delays gastric emptying in humans with direct effects on glycemic control. Cell Metab. 27:1201–1211.e3. 10.1016/j.cmet.2018.04.008 29731416

[phy213788-bib-0019] Orgaard, A. , and J. J. Holst . 2017 The role of somatostatin in GLP‐1‐induced inhibition of glucagon secretion in mice. Diabetologia 60:1731–1739. 28551699 10.1007/s00125-017-4315-2 PMC5552842

[phy213788-bib-0020] Orskov, C. , J. Jeppesen , S. Madsbad , and J. J. Holst . 1991 Proglucagon products in plasma of noninsulin‐dependent diabetics and nondiabetic controls in the fasting state and after oral glucose and intravenous arginine. J. Clin. Invest. 87:415–423. 1991827 10.1172/JCI115012 PMC295092

[phy213788-bib-0021] Ørskov, C. , L. Rabenhøj , A. Wettergren , H. Kofod , and J. J. Holst . 1994 Tissue and plasma concentrations of amidated and glycine‐extended glucagon‐like peptide I in humans. Diabetes 43:535–539. 8138058 10.2337/diab.43.4.535

[phy213788-bib-0022] Ostrowski, K. , T. Rohde , M. Zacho , S. Asp , and B. K. Pedersen . 1998 Evidence that interleukin‐6 is produced in human skeletal muscle during prolonged running. J. Physiol. 508(pt 3):949–953. 9518745 10.1111/j.1469-7793.1998.949bp.x PMC2230908

[phy213788-bib-0023] Pedersen, B. K. , A. Steensberg , and P. Schjerling . 2001 Muscle‐derived interleukin‐6: possible biological effects. J. Physiol. 536(Pt 2):329–337. 11600669 10.1111/j.1469-7793.2001.0329c.xd PMC2278876

[phy213788-bib-0024] Scheller, J. , A. Chalaris , D. Schmidt‐Arras , and S. Rose‐John . 2011 The pro‐and anti‐inflammatory properties of the cytokine interleukin‐6. Biochim. Biophys. Acta 1813:878–888. 21296109 10.1016/j.bbamcr.2011.01.034

[phy213788-bib-0025] Steensberg, A. , G. Hall , T. Osada , M. Sacchetti , B. Saltin , and B. K. Pedersen . 2000 Production of interleukin‐6 in contracting human skeletal muscles can account for the exercise‐induced increase in plasma interleukin‐6. J. Physiol. 529:237–242. 11080265 10.1111/j.1469-7793.2000.00237.x PMC2270169

[phy213788-bib-0026] Steensberg, A. , C. P. Fischer , C. Keller , K. Møller , and B. K. Pedersen . 2003a IL‐6 enhances plasma IL‐1ra, IL‐10, and cortisol in humans. Am. J. Physiol. Endocrinol. Metab. 285:E433–E437. 12857678 10.1152/ajpendo.00074.2003

[phy213788-bib-0027] Steensberg, A. , C. P. Fischer , M. Sacchetti , C. Keller , T. Osada , P. Schjerling , et al. 2003b Acute interleukin‐6 administration does not impair muscle glucose uptake or whole‐body glucose disposal in healthy humans. J. Physiol. 548:631–638. 12640021 10.1113/jphysiol.2002.032938 PMC2342867

[phy213788-bib-0028] Svendsen, B. , R. Pais , M. S. Engelstoft , N. B. Milev , P. Richards , C. B. Christiansen , et al. 2016 GLP1‐and GIP‐producing cells rarely overlap and differ by bombesin receptor‐2 expression and responsiveness. J. Endocrinol. 228:39–48. 26483393 10.1530/JOE-15-0247 PMC7212066

[phy213788-bib-0029] Timper, K. , E. Dalmas , E. Dror , S. Rütti , C. Thienel , N. S. Sauter , et al. 2016 Glucose‐dependent insulinotropic peptide stimulates glucagon‐like peptide 1 production by pancreatic islets via interleukin 6, produced by alpha cells. Gastroenterology 151:165–179. 26971825 10.1053/j.gastro.2016.03.003

[phy213788-bib-0030] Vilsboll, T. , and J. J. Holst . 2004 Incretins, insulin secretion and Type 2 diabetes mellitus. Diabetologia 47:357–366. 14968296 10.1007/s00125-004-1342-6

[phy213788-bib-0001] Wewer Albrechtsen, N. J. , M. J. Bak , B. Hartmann , L. W. Christensen , R. E. Kuhre , C. F. Deacon , et al. 2015 Stability of glucagon‐like peptide‐1 and glucagon in human plasma. Endocr. Connect. 4:50–57. 25596009 10.1530/EC-14-0126 PMC4317691

[phy213788-bib-0031] Windeløv, J. A. , N. J. Albrechtsen , R. E. Kuhre , S. L. Jepsen , D. Hornburg , J. Pedersen , et al. 2017 Why is it so difficult to measure glucagon‐like peptide‐1 in a mouse? Diabetologia 60:2066–2075. 28669086 10.1007/s00125-017-4347-7

[phy213788-bib-0032] Wueest, S. , C. I. Laesser , M. Böni‐Schnetzler , F. C. Lucchini , M. Borsigova , W. Müller , et al. 2018 IL‐6–type cytokine signaling in adipocytes induces intestinal GLP‐1 secretion. Diabetes 67:36–45. 29066599 10.2337/db17-0637

[phy213788-bib-0033] Xu, H. , G. T. Barnes , Q. Yang , G. Tan , D. Yang , C. J. Chou , et al. 2003 Chronic inflammation in fat plays a crucial role in the development of obesity‐related insulin resistance. J. Clin. Invest. 112:1821–1830. 14679177 10.1172/JCI19451 PMC296998

